# Public Awareness and Perceptions of Stem Cell Therapy for Stroke: A Cross-Sectional Survey Study

**DOI:** 10.7759/cureus.115784

**Published:** 2026-09-04

**Authors:** Shreya P Kumar, Prasanna V Kumar

**Affiliations:** 1 Medical Education, Smithtown High School East, Smithtown, USA; 2 Medicine/Cardiology, New York University (NYU) Langone Health, New York, USA

**Keywords:** clinical trials, health literacy, ischemic stroke, public awareness, regenerative medicine, stem cell therapy, survey

## Abstract

Background

Stroke remains a leading cause of death and long-term disability. Although stem cell-based approaches are being investigated as possible regenerative strategies for stroke, public perceptions of their potential benefits, risks, mechanisms, and clinical use are not well-characterized. This exploratory survey described responses to an investigator-developed questionnaire about stem cell therapy for stroke.

Methods

A 10-item cross-sectional questionnaire was distributed electronically through Google Forms (Google, Inc., Mountain View, CA) to 36 individuals in a convenience sample; 26 completed questionnaires were received (response rate: 72.2%). The questionnaire was investigator-developed and was not validated as a measure of factual knowledge, clinical efficacy, or safety. Responses were categorized as “yes,” “no,” or “maybe”; one item used time-based response categories. Counts and percentages were calculated using the total number of completed questionnaires as the denominator. The survey was independently conducted as a student-led summer academic project and did not undergo review by an institutional review board, school ethics committee, or independent ethics authority.

Results

Twenty-six responses were analyzed. For the item asking whether embryonic stem cells are effective in stroke treatment, eight respondents (30.77%) selected “yes,” 16 (61.54%) selected “maybe,” and two (7.69%) selected “no.” For the item asking whether stem cell treatment has advantages for stroke, 21 respondents (80.77%) selected “yes,” four (15.38%) selected “maybe,” and one (3.85%) selected “no.” “Maybe” was the majority or joint-most-frequent response for six of 10 items, including items about animal testing, adverse effects, alternative regenerative treatments, and use during pregnancy.

Conclusions

In this small convenience sample, responses to an investigator-developed questionnaire suggested favorable perceptions of possible benefits of stem cell therapy for stroke, alongside frequent uncertainty about safety, mechanisms, the timing of improvement, and use in special populations. Because the questionnaire was not validated as a factual-knowledge instrument, findings should be interpreted strictly as descriptive perceptions rather than as evidence of respondents’ medical knowledge, treatment confidence, or the clinical efficacy or safety of stem cell therapy. Larger studies using validated instruments and representative sampling are needed.

## Introduction

Stroke is a major cause of death and long-term disability. Ischemic stroke results from the occlusion of a cerebral artery, whereas hemorrhagic stroke results from the rupture of a cerebral blood vessel. Prompt recognition and treatment are essential because acute ischemic stroke therapies are time-sensitive [1].

Acute ischemic stroke therapies, including intravenous thrombolysis and endovascular thrombectomy for eligible patients, aim to restore cerebral perfusion and reduce disability. Poststroke rehabilitation seeks to improve function and independence after neurological injury; however, these approaches do not constitute established regenerative therapies that replace infarcted neural tissue [2,3].

Stem cells are undifferentiated cells with the capacity for self-renewal and differentiation. Embryonic stem cells and induced pluripotent stem cells are pluripotent, whereas adult tissue-specific stem cells have more restricted developmental potential [4,5]. In preclinical stroke studies, mesenchymal stem cells have been associated with immunomodulation, the release of trophic factors and extracellular vesicles, the modulation of angiogenesis and neurogenesis, and the support of neural remodeling [6-9]. The relative importance of these proposed mechanisms varies by cell type, experimental model, the route of administration, and outcome measure. Direct neuronal replacement is not established as the predominant mechanism of mesenchymal stem cell therapy for stroke [6-9].

Cell-based interventions raise questions about safety, dosing, cell type, delivery route, and clinical effectiveness. Stem cell therapies for stroke have been studied predominantly in preclinical models and early-phase human trials. A systematic review of preclinical and clinical mesenchymal stem cell studies concluded that further rigorous clinical evaluation is needed. The Pilot Investigation of Stem Cells in Stroke (PISCES) trial evaluated the intracerebral implantation of a conditionally immortalized human neural stem cell line in patients with chronic ischemic stroke disability and reported feasibility and tolerability in a phase 1 study [10]. PISCES III was designed as a randomized, sham-controlled trial to evaluate efficacy more rigorously. Stem cell-based therapies are not established as routine regenerative treatment or curative therapy for ischemic stroke.

Understanding public perceptions of potential regenerative therapies may be relevant to future patient education, informed-consent communication, and clinical-trial design. Prior studies suggest that favorable attitudes toward stem cell therapy may coexist with limited baseline knowledge. In a cohort of ischemic stroke survivors, 12% reported prior knowledge of stem cell therapy, although 63% expressed positive attitudes after receiving standardized information. A systematic review found variable patient and public understanding of cell and gene therapies and highlighted the need for accessible education regarding potential benefits, risks, and treatment uncertainty.

The aim of this exploratory, student-led cross-sectional survey was to describe responses to an investigator-developed questionnaire regarding perceptions of embryonic stem cell therapy for stroke among a convenience sample in Smithtown, New York. The prespecified descriptive outcomes were the frequencies and percentages of each response category for the 10 questionnaire items. The survey was not designed or validated to determine factual medical knowledge, clinical efficacy, treatment safety, regulatory approval, or the appropriateness of treatment in special populations.

## Materials and methods

Study design and setting

This was a descriptive, cross-sectional pilot survey conducted independently as a student-led summer academic project. The investigator-developed questionnaire was created using Google Forms (Google, Inc., Mountain View, CA) and distributed electronically through family, friends, and a school-associated network in Smithtown, New York, during summer 2025.

Participants and sampling

Participants were recruited using convenience sampling. Individuals who received the survey link and voluntarily completed the questionnaire were included. No incentives were offered. The survey link was distributed electronically through the student investigator’s family, friends, and school-associated network in Smithtown, New York. The accompanying invitation described the activity as a research project intended to learn about the awareness of stem cells in stroke treatment, invited recipients to complete a brief survey, and stated that completion would take approximately one minute or less. The link was sent to 36 individuals, and 26 completed questionnaires were received, yielding a response rate of 72.2% (26/36).

All 26 submitted questionnaires contained responses to all 10 survey items and were included in the descriptive analysis. No questionnaires were excluded because of missing item responses. Because the survey was anonymous and no identifying information, IP addresses, or other tracking data were collected, duplicate participation and participant eligibility could not be independently verified. No names, contact information, demographic characteristics, medical history, stroke history, or other directly identifying information was collected.

Questionnaire development and data collection

The 10-item questionnaire was investigator-developed by the student investigator for this summer academic project. No published, standardized, or validated questionnaire was used, and the instrument was not copied from or adapted from a validated medical-knowledge scale. The questions were generated to explore broad perceptions of possible effectiveness, benefits, adverse effects, mechanisms, the timing of improvement, and the possible use of embryonic stem cell therapy for stroke in selected populations.

No formal expert review, pilot testing, content-validity assessment, reliability testing, or psychometric validation was performed. The questionnaire was designed to describe responses and perceptions; it was not designed to classify answers as medically correct or incorrect, measure factual knowledge, assess health literacy, establish clinical efficacy or safety, or assess the awareness of the current evidence base. Nine items used “yes,” “no,” or “maybe” response options. One item used “few hours,” “few days,” or “few weeks.” The complete questionnaire is presented in Table 1.

**Table 1:** Investigator-developed questionnaire assessing perceptions of stem cell therapy for stroke. Response options: items 1-3, 5, 6, and 8-10: yes, maybe, or no; item 4: few hours, few days, or few weeks; item 7: true, maybe, or false.

Item	Survey question
1	Are embryonic stem cells effective in the treatment of stroke?
2	Is clinical treatment, including aspirin, thrombolytics, interventions, physical therapy, and rehabilitation, the only method to treat stroke?
3	Is testing embryonic stem cells in mice the only way to determine efficacy? Has this been done in stroke models?
4	How long does it take for symptoms to improve after embryonic stem cell injection?
5	Are there adverse effects associated with using stem cells for stroke?
6	Are there advantages to using stem cell treatment for stroke?
7	Embryonic stem cells play a major role in curing stroke because they help regenerate neurons.
8	Is there another treatment, besides stem cells, that helps regenerate neurons after stroke?
9	Can younger children receive this treatment?
10	Can stem cells be used in pregnant women?

No separate formal participant information or consent statement was displayed on the first page of the Google Forms before the questionnaire. Participation was voluntary and anonymous, no compensation was offered, and the completion of the questionnaire was treated as implied consent.

Data type and statistical analysis

The primary data were categorical survey responses. Responses were reviewed manually, and frequencies and percentages were calculated manually for each response option using the total number of completed questionnaires as the denominator (n=26). Descriptive statistics only were used. No hypothesis tests, regression analyses, or adjusted analyses were performed because this was an exploratory pilot survey with a small convenience sample and no participant-level demographic or clinical covariates. Potential confounders, including age, sex, education, health literacy, prior exposure to stem cell information, and the awareness of available evidence, were not measured and therefore could not be controlled for.

Sample-size consideration

No formal a priori sample-size calculation was performed. The final sample of 26 completed questionnaires was a convenience sample and was not intended to provide population-representative estimates.

Ethics

This exploratory survey was independently initiated and conducted by the student investigator during her summer break as a student academic project under parental supervision. The project was not conducted under the auspices of, sponsored by, funded by, or affiliated with a school, university, hospital, clinic, or other research institution. No institutional review board, school ethics committee, or independent ethics authority reviewed the study or issued a formal exemption or determination regarding the need for ethics review.

Participation was voluntary and anonymous. The survey invitation stated that the activity was a research project about the awareness of stem cells in stroke treatment and invited recipients to complete a brief questionnaire. No separate formal participant information or consent statement was displayed before the questionnaire. The survey involved no intervention, clinical procedure, collection of biological specimens, access to medical records, protected health information, or directly identifying information. No compensation was offered. The completion of the anonymous voluntary questionnaire was treated as implied consent to participate. The absence of prospective formal ethics review is acknowledged as a limitation of this exploratory project.

## Results

Table 2 summarizes the response distribution for all 10 survey items. For the item asking whether embryonic stem cells are effective in stroke treatment, eight respondents (30.77%) selected “yes,” 16 (61.54%) selected “maybe,” and two (7.69%) selected “no.” For the item asking whether conventional clinical treatment is the only approach to stroke treatment, 13 respondents (50.00%) selected “no,” eight (30.77%) selected “maybe,” and five (19.23%) selected “yes.”

**Table 2:** Distribution of responses to the 10-item survey on perceptions of stem cell therapy for stroke (n=26). Question wording is provided in Table 1. Each question has three response categories; percentages are calculated using n=26 as the denominator.

Question number	Response option	Number of respondents	Percentage
1	Yes	8	30.77%
1	Maybe	16	61.54%
1	No	2	7.69%
2	Yes	5	19.23%
2	Maybe	8	30.77%
2	No	13	50.00%
3	Yes	4	15.38%
3	Maybe	11	42.31%
3	No	11	42.31%
4	Few hours	3	11.54%
4	Few days	10	38.46%
4	Few weeks	13	50.00%
5	Yes	7	26.92%
5	Maybe	12	46.15%
5	No	7	26.92%
6	Yes	21	80.77%
6	Maybe	4	15.38%
6	No	1	3.85%
7	True	16	61.54%
7	Maybe	7	26.92%
7	False	3	11.54%
8	Yes	6	23.08%
8	Maybe	11	42.31%
8	No	9	34.62%
9	Yes	14	53.85%
9	Maybe	8	30.77%
9	No	4	15.38%
10	Yes	5	19.23%
10	Maybe	11	42.31%
10	No	10	38.46%

For the item on animal testing, 11 respondents (42.31%) selected “no,” 11 (42.31%) selected “maybe,” and four (15.38%) selected “yes.” Thirteen respondents (50.00%) selected “few weeks” as the expected time for symptom improvement after stem cell injection, 10 (38.46%) selected “few days,” and three (11.54%) selected “few hours.”

For the item concerning adverse effects of stem cell use for stroke, 12 respondents (46.15%) selected “maybe,” while seven respondents (26.92%) selected “yes,” and seven (26.92%) selected “no.” For the item asking whether stem cell treatment has advantages for stroke, 21 respondents (80.77%) selected “yes,” four (15.38%) selected “maybe,” and one (3.85%) selected “no.” For the statement that embryonic stem cells play a major role in curing stroke because they help regenerate neurons, 16 respondents (61.54%) selected “true,” seven (26.92%) selected “maybe,” and three (11.54%) selected “false.”

For the item asking whether another treatment besides stem cells helps regenerate neurons after stroke, 11 respondents (42.31%) selected “maybe,” nine (34.62%) selected “no,” and six (23.08%) selected “yes.” For the item asking whether younger children can receive stem cell treatment, 14 respondents (53.85%) selected “yes,” eight (30.77%) selected “maybe,” and four (15.38%) selected “no.” For the item asking whether stem cells can be used in pregnant women, five respondents (19.23%) selected “yes,” 11 (42.31%) selected “maybe,” and 10 (38.46%) selected “no.”

These findings are presented descriptively. The response distributions do not establish whether individual responses reflect factual medical knowledge, clinical understanding, treatment confidence, clinical efficacy, treatment safety, regulatory approval, or appropriate treatment use in any population.

## Discussion

This student-led pilot survey describes responses to an investigator-developed questionnaire about embryonic stem cell therapy for stroke in a small convenience sample. Responses to items addressing potential benefit were frequently favorable, while “maybe” was common across multiple items concerning efficacy, adverse effects, mechanisms, time to improvement, alternative regenerative approaches, and use in children or pregnant women. Because the questionnaire was not validated as a measure of factual knowledge, these response patterns should be interpreted as perceptions within this sample rather than as evidence of correct or incorrect medical knowledge, the awareness of the current evidence base, or confidence in treatment efficacy.

Potential interest in stem cell therapy may relate to the substantial unmet need for approaches that improve recovery after neurological injury. Acute ischemic stroke treatment seeks to restore blood flow promptly in eligible patients, and rehabilitation supports the recovery of function, but neither is an established regenerative intervention that replaces lost brain tissue [2,3]. Preclinical literature suggests that mesenchymal stem cells may influence inflammation, angiogenesis, and neural-repair processes through paracrine and immunomodulatory mechanisms [6-9]. These proposed mechanisms vary across cell types, models, and treatment approaches and should not be equated with proven clinical neuronal replacement, established clinical benefit, or a cure for stroke in humans.

The questionnaire included items regarding potential adverse effects and possible use in children and pregnant women. These items were intended to describe respondent perceptions and were not intended to imply that embryonic stem cell therapy is approved, established, safe, or recommended for these populations. The survey did not assess respondents’ knowledge of the evidence, treatment safety, regulatory status, or clinical appropriateness in special populations. Early-phase human studies, including PISCES, have examined feasibility and safety in selected adults with chronic ischemic stroke [10,11]. More rigorous trials with clinically meaningful outcomes, appropriate comparator groups, and longer follow-up are necessary to define potential benefits and harms [9,12].

Prior research has reported variable public and patient familiarity with cell and gene therapies and has identified a need for accessible communication regarding potential benefits, risks, uncertainty, and the distinction between experimental research and established clinical care [13,14]. In the United States, no FDA-approved stem cell product is indicated to treat stroke, and regenerative medicine products marketed for stroke treatment should not be presumed to be approved or proven safe or effective [15]. The uncertainty observed in the present survey may be related to several unmeasured factors, including variation in prior exposure to stem cell information and the awareness of the available evidence. However, this study did not assess those factors and cannot determine the cause of any observed uncertainty.

Limitations

This study has important limitations. The sample size was small, recruitment used convenience sampling, and the survey was distributed through family, friends, and a school-associated network in one geographic area. Of the 36 individuals who received the survey link, 26 completed the questionnaire, yielding a response rate of 72.2%. The findings are not generalizable to the broader public, stroke survivors, pregnant individuals, children, or patients considering clinical trials.

No demographic or clinical variables were collected. Thus, differences related to age, sex, education, health literacy, prior familiarity with stem cell research, personal experience with stroke, or the awareness of the available evidence could not be assessed or controlled for. The questionnaire was investigator-developed and did not undergo formal expert review, pilot testing, content-validity assessment, reliability testing, or psychometric validation. Several items used potentially leading or clinically imprecise language, including statements about “curing” stroke, neuron regeneration, the expected timing of improvement, and possible use in children and pregnant women.

Accordingly, responses cannot be classified as correct or incorrect medical knowledge and should not be interpreted as evidence of treatment confidence, health literacy, clinical efficacy, treatment safety, regulatory approval, or the appropriateness of embryonic stem cell therapy in special populations. The survey did not measure participants’ prior knowledge or awareness of the available evidence; therefore, it cannot establish the causes of observed uncertainty. The absence of prospective review by an institutional review board or independent ethics authority is an additional limitation. Although the survey was voluntary and anonymous and did not collect clinical or directly identifying information, future surveys involving human participants should be conducted with appropriate prospective institutional ethics oversight or a formal determination, as applicable.

## Conclusions

In this exploratory, student-led cross-sectional survey, a small convenience sample provided responses indicating favorable perceptions of possible benefits of embryonic stem cell therapy for stroke, accompanied by frequent uncertainty across several related items. The questionnaire was investigator-developed and not validated as a factual-knowledge or clinical-efficacy assessment; therefore, these results describe perceptions only and do not establish respondents’ medical knowledge, treatment confidence, or the efficacy, safety, approval status, or appropriate clinical use of stem cell therapy.

Future research should use larger, more representative samples and validated instruments that clearly distinguish knowledge, attitudes, perceptions, and willingness to participate in research. Independently of these survey findings, well-designed clinical trials remain necessary to define the safety and efficacy of stem cell-based interventions for ischemic stroke.
